# Midwifery and General Practice

**Published:** 1899-03-25

**Authors:** 


### MIDWIFERY AND GENERAL PRACTICE.

The midwives evidently intend to carry the war into the enemy's country, and to smite the general practitioner hip and thigh. Here is their manifesto, which appears in the form of a notice on an agenda paper of the Midwives' Society: "That as the practice of medicine and surgery together with the practice of midwifery, as is customary by a very large proportion of the members of the medical profession and by many district nurses, is provocative of grave danger and death to lying-in women, to petition Parliament to stop such heterogeneous work and compel all who attend lying-in women for gain to become and remain purely midwifery practitioners." That is to say, that if a male practitioner presumes to undertake midwifery, he must make himself a man-midwife and nothing else. Of course, this is a return stroke in answer to the charge that if the midwives once became "qualified" they would not content themselves with midwifery, but would soon begin to do a little quacking in medicine and surgery. This little resolution clearly says to the doctors "if we can't do medicine you can't do midwifery." But these ingenious ladies forget that when a doctor does midwifery he does that for which he is specially qualified according to law, while when they treat disease the position is precisely the reverse.

